# Novel Downhole Electromagnetic Flowmeter for Oil-Water Two-Phase Flow in High-Water-Cut Oil-Producing Wells

**DOI:** 10.3390/s16101703

**Published:** 2016-10-14

**Authors:** Yanjun Wang, Haoyu Li, Xingbin Liu, Yuhui Zhang, Ronghua Xie, Chunhui Huang, Jinhai Hu, Gang Deng

**Affiliations:** 1 Harbin Institute of Technology, School of Electrical Engineering and Automation, Harbin 150001, China; dlts_wangyj@petrochina.com.cn; 2 Daqing Oilfield Limited Company, Daqing 163412, China; dlts_zhangyhui@petrochina.com.cn (Y.Z.); xieronghua@petrochina.com.cn (R.X.); dlts_huangch@petrochina.com.cn (C.H.); dlts_hujh@petrochina.com.cn (J.H.); denggang@petrochina.com.cn (G.D.)

**Keywords:** electromagnetic flowmeter, weight function, magnetic field, oil-water two-phase flow, flowrate measurement, oil-producing well, onsite experiments

## Abstract

First, the measuring principle, the weight function, and the magnetic field of the novel downhole inserted electromagnetic flowmeter (EMF) are described. Second, the basic design of the EMF is described. Third, the dynamic experiments of two EMFs in oil-water two-phase flow are carried out. The experimental errors are analyzed in detail. The experimental results show that the maximum absolute value of the full-scale errors is better than 5%, the total flowrate is 5–60 m^3^/d, and the water-cut is higher than 60%. The maximum absolute value of the full-scale errors is better than 7%, the total flowrate is 2–60 m^3^/d, and the water-cut is higher than 70%. Finally, onsite experiments in high-water-cut oil-producing wells are conducted, and the possible reasons for the errors in the onsite experiments are analyzed. It is found that the EMF can provide an effective technology for measuring downhole oil-water two-phase flow.

## 1. Introduction

Production profile logging is a significant methodology in oilfield development, which can provide crucial information—such as oil-water output, temperature, and pressure—to evaluate reservoirs and identify high-aquifer potential through the placement of logging instruments in oil wells. Production profile logging is also useful for optimizing reservoir engineering plans and evaluating fracturing and water plugging effects. Over the past 30 years, the turbine flowmeter has been widely used for the production profile logging at Daqing Oilfield. In recent years, with the applications of enhanced oil recovery technologies, such as mass fracturing and chemical flooding, the flow of fluid in oil wells is much more complicated with fluids carrying sand and solid impurity concentration in the wellbore. Turbine flowmeters are frequently blocked by the rotating blades during logging, leading to measurement failure. Therefore, it is of important practical significance to develop downhole flowrate measurement technology with no moving parts and high reliability to further improve the success rate of well logging. The EMF is widely used to measure the injection rate in polymer-injection wells at Daqing Oilfield and is unaffected by temperature, viscosity, density, pressure, gravity, and other physical parameters of the liquid [1]. Inspired by successful utilization of EMF in polymer-injection wells, it is reasonable to believe that oil-water two-phase flow can be measured by the EMF in high-water-cut oil-producing wells.

Shercliff [2] first developed the theory for the voltage-sensing EMF and computed the weight function that represents the contribution of the fluid velocity to the signal in the cross-section of a conduit for a two-dimensional single-phase flow. Wyatt [3] analytically calculated the weight function as a series solution for annular and single-phase flows using the two-dimensional flowmeter equation and then described the weight function distribution of the annular flowmeter with a coaxial insulating core for a two-phase flow. Zhang [4] theoretically solved the two-dimensional weight function of flowmeters in both concentric and eccentric annular flows by simplifying a gas-liquid two-phase flow as annular flow. Zhang [5] also investigated the effect of phase distribution on the weight function in a two-dimensional annular domain with and without eccentricity, proving that the weight functions of multi-pair electrodes and uniform-field electromagnetic flowmeters with or without eccentricity were series solutions. Wang et al. [6] introduced the theory of the magnetic dipole and deduced the magnetic field distribution formula of a single magnetic dipole. Wang et al. [7] investigated the relationship between the induced electric potential and velocity distribution of the conductive continuous phase in two-phase flows in pipes to which an electromagnetic field is applied; the numerical simulation results suggested that electromagnetic flow metering may be an effective novel method for measuring the axial velocity profile of the conducting continuous phase.

Tewodros [8] designed a capacitive-type EMF, and the experimental result clearly indicated that it can be used in both dielectric liquids and conductive liquids. Wang et al. [9] developed a capacitive EMF working in voltage sensing mode, providing better performance than the electrode type flowmeter for oil-water two-phase volumetric flowrate measurement. Bernier et al. [10] investigated the transverse field EMF for two-phase flows, which can monitor the average liquid velocity independent of void fraction, flowrate, or flow regime. Ishii et al. [11] carried out extensive experiments on the vertical slug flow by an EMF and an impedance void-meter in an air-water two-phase experimental loop. For the first time, the area-averaged liquid velocity of slug flow was revealed by the EMF. Cha et al. [12] investigated the characteristics of the EMF in a water-air mixture and a liquid sodium-nitrogen mixture, encompassing bubbly to slug flow regimes; the EMF exhibited good potential as a useful device for identifying the flow regimes. 

Hemp et al. [13] and Rosales et al. [14,15] described problems in the theory and designed EMFs for dielectric liquids, and many efforts were devoted to experimental measurements of electrode signal noise spectra in the dielectric liquid BP180, an analytical model of the generation of noise by turbulence modulation of the background charge distribution, and an analytical model of the generation of noise by charged particles. In addition, Cui et al. [16] proposed a novel method for multi-information acquisition from the EMF, using magnetic excitation to measure the fluid velocity and electrochemistry impedance spectroscopy for both the fluid quality and the contamination level of the transducer. Stelian [17] used numerical modeling to calibrate a Lorentz force flowmeter in different flow conditions. The research results showed that the velocity distribution in laminar flow should be included in the numerical calibration procedure while numerical calibration can be performed for a solid conductor in turbulent flows. Laogun et al. [18] measured phase delays between flow velocity pulses at the proximal and distal ends of two arterial segments in dogs under two vasoactive conditions (vasoconstriction and vasodilation) by the EMF and investigated the variation of phase velocity with changing frequency in the respective segments.

It is worth emphasizing that there is no literature reporting the use of the downhole EMF to measure the flowrate of oil-water two-phase flow in oil wells. In this work, we proposed a novel downhole inserted EMF for flowrate measurement of oil-water two-phase flow in high-water-cut oil-producing wells. The ideal response model of the downhole inserted two-electrode EMF was established, and the weight function and intensity of the magnetic field were derived and calculated. Furthermore, the dynamic experiments of oil-water two-phase flow were conducted by using the EMF, and the details of the measurement errors were discussed. Finally, the onsite experiments were analyzed by using the EMF.

## 2. Theory of the Downhole Two-Electrode EMF

### 2.1. Ideal Response Model of the Two-Electrode EMF

**Figure 1:**
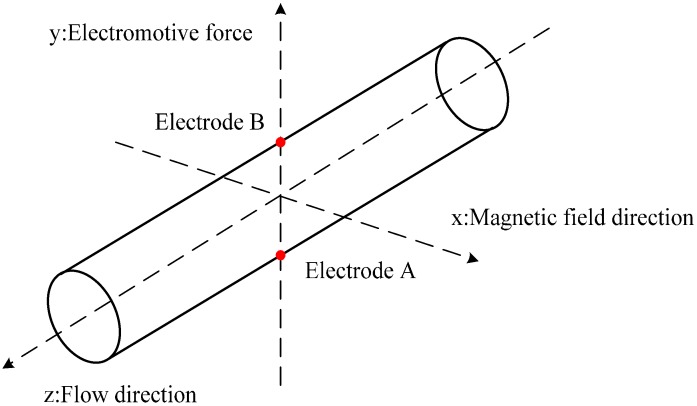
Schematic diagram of the uniform magnetic field EMF.

Based on the assumption of a uniform magnetic field and a rectilinear flow, the conventional form of the EMF is shown in Figure 1. The distribution of the potential *U* inside the flowtube of the EMF is described by the following basic equation:

(1) $$\nabla^{2}U=\nabla \cdot \left(v\times B\right)$$

where ***v*** and ***B*** are the velocity vector and the magnetic flux density vector, respectively.

**Figure 2:**
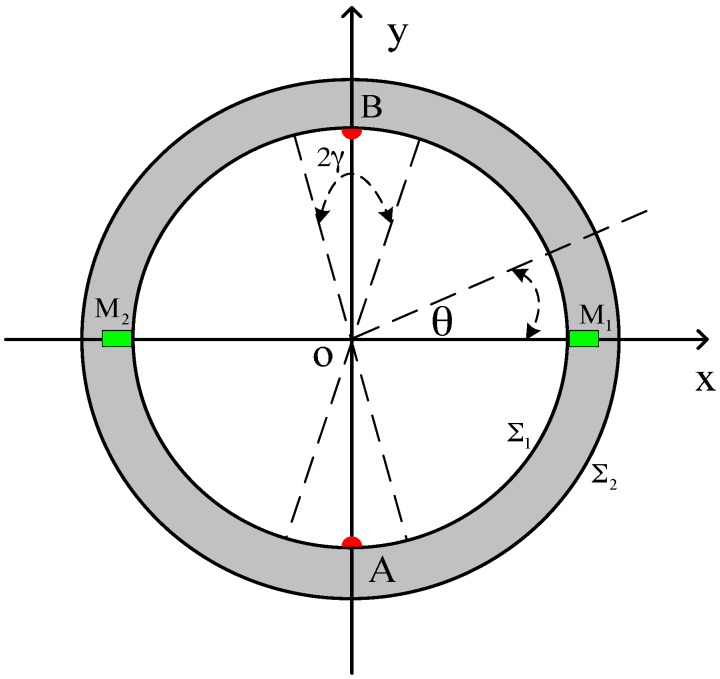
Cross section top view of the downhole inserted EMF.

Figure 2 shows a schematic diagram of the downhole inserted two-electrode EMF, A and B represent two electrodes placed on the inner wall at intervals of 180° inside the flowtube, *M*_1_ and *M*_2_ represent two magnetic dipoles inlaid on the wall of the flowtube at intervals of 180°, and $\sum_{1}$ and $\sum_{2}$ represent the inner and outer surfaces of the EMF, respectively. 

To simplify the mathematical expressions, we start with the extreme case of a two-dimensional model, for which we give detailed expressions for the solution and discuss the results.

The response equation of the downhole inserted two-electrode EMF can be obtained by solving partial differential Equation (1). Using the Green function *G* to solve Equation (1), where *G* satisfies Laplace’s equation on the inner surface of the flowmeter:

(2) $$\nabla^{2}G=0$$

where ∇ is the gradient operator, and $\nabla^{2}$ is the Laplace operator.

The boundary condition on the inner surface of the flowmeter in this situation is given by:

(3) $$\frac{\partial U}{\partial n}=0$$

where *n* is the normal of the boundary.

That is,

(4) $$\frac{\partial G}{\partial n}=\left\{ \begin{array}{cc} +\frac{\pi aL}{S_{0}} & \mathrm{at}\;r=a,\mathrm{on}\;\mathrm{the}\;\mathrm{electrode}\;A\;\\ -\frac{\pi aL}{S_{0}} & \mathrm{at}\;r=a,\mathrm{on}\;\mathrm{the}\;\mathrm{electrode}\;B\;\\ \;0 & \mathrm{at}\;r=a,\;\mathrm{otherwise}\; \end{array}\right.$$

where *S*_0_ is the area of the electrode, *a* is the inner radius, and *L* is the half-length of the flowmeter.

The identity

(5) $$U\nabla^{2}G-G\nabla^{2}U=\nabla \cdot \left(U\nabla G-G\nabla U\right)$$

is used to solve the internal volume integral of the flowtube, and the volume integral can be transformed into a surface integral using Gauss’ theorem by applying the following equation:

(6) $$\int \nabla \cdot FdV=\oint F_{n}d\Sigma$$

where ***F*** is the arbitrary vector function, d*V* is the volume element, dΣ is the boundary area element, and ∮d∑ is the surface integral around the entire boundary.

Substituting Equation (5) into Equation (6) yields

(7) $$\int \left(U\nabla^{2}G-G\nabla^{2}U\right)dV=\oint \left(U\frac{\partial G}{\partial n}-G\frac{\partial U}{\partial n}\right)d\sum$$

By substituting Equations (1)–(4) into Equation (7), the induced potential difference can be given by:

(8) $$U_{AB}=\frac{1}{\pi aL}\int v\cdot \left(B\times \nabla G\right)dV$$

where ***B*** is the magnetic induction intensity, and ∇G is referred to as the weight function that can be simplified as ***W***.

Equation (8) is the general formula used to determine the induced potential difference of the downhole inserted two-electrode EMF. In the two-dimensional situation, the induced potential difference can be expressed in polar coordinates as follows:

(9) $$U_{AB}=\frac{1}{\pi aL}\int_{0}^{a}v\cdot \left(B\times W\right)rdr\int_{0}^{2\pi }d\theta$$

where *a* represents the inner radius of the EMF.

### 2.2. Weight Function of the Two-Electrode EMF

The weight function ***W*** represents the contribution of the fluid velocity to the induced potential difference. In fact, even under the same velocity and magnetic field conditions, given that the distances and orientations between fluid units and electrodes are different in the measurement area, the contribution of the fluid units to the induced potential difference remains different, i.e., ***W*** has larger values near the electrodes and vice versa.

To solve the expression of the weight function in the cylindrical coordinate system, we suppose that the electrodes are linear, have the same length as the flowmeter, and possess central angles 2γ, as shown in Figure 2. For the linear electrodes, suppose γ→0, such that $S_{0}=4\gamma aL$ in Equation (4) can be written as follows:

(10) $$\frac{\partial G}{\partial n}=\left\{ \begin{array}{ll} +\frac{\pi }{4\gamma } & \mathrm{at}\;r=a,\mathrm{on}\;\mathrm{the}\;\mathrm{electrode}\;A\;\\ -\frac{\pi }{4\gamma } & \mathrm{at}\;r=a,\mathrm{on}\;\mathrm{the}\;\mathrm{electrode}\;B\;\\ 0 & \mathrm{at}\;r=a,\;\mathrm{otherwise}\; \end{array}\right.$$

By using the segregation variable method, *G* can be expressed as:

(11) $$G=\sum_{n=0}^{\infty }\left(C_{1}r^{n}+C_{1}^{\prime }r^{-n}\right)\left[C_{n}\sin\left(n\theta \right)+C_{n}^{\prime }\cos\left(n\theta \right)\right]$$

We can easily verify that the formula satisfies Laplace’s equation by substituting Equation (11) into Equation (2). Furthermore, to determine the coefficients $C_{1}$, $C_{1}^{\prime }$, $C_{n}$, and $C_{n}^{\prime }$ in Equation (11), we employ the solution below.

Suppose

(12) $$R=C_{1}r^{n}+C_{1}^{\prime }r^{-n}$$

when r→0 and $r^{n}\to \infty$, $C_{1}^{\prime }=0$. We derive the special solution of *G*:

(13) $$G=\sum_{n=0}^{\infty }r^{n}[C_{n}\sin(n\theta )+C_{n}^{\prime }\cos(n\theta )]$$

where the values of $C_{n}$ and $C_{n}^{\prime }$ are different from those in Equation (11).

The surface $\sum_{1}$ shown in Figure 2 is the inner surface of the flowmeter that satisfies the following boundary condition:

$$\frac{\partial G}{\partial n}={\frac{\partial G}{\partial r}|}_{r=a}=\left\{ \begin{array}{lll} \frac{\pi }{4\gamma } & \mathrm{on}\;\mathrm{the}\;\mathrm{electrode}\;A, & \theta \in \left[\frac{3\pi }{2}-\gamma ,\;\frac{3\pi }{2}+\gamma \right]\\ -\frac{\pi }{4\gamma } & \mathrm{on}\;\mathrm{the}\;\mathrm{electrode}\;B, & \theta \in \left[\frac{\pi }{2}-\gamma ,\;\frac{\pi }{2}+\gamma \right]\\ 0 & \mathrm{otherwise} & \end{array}\right.$$

which can be written as follows:

(14) $$\sum_{n=0}^{\infty }na^{n-1}[C_{n}\sin(n\theta )+{C_{n}}^{\prime }\cos(n\theta )]=\left\{ \begin{array}{lll} \frac{\pi }{4\gamma } & \mathrm{on}\;\mathrm{the}\;\mathrm{electrode}\;A, & \theta \in \left[\frac{3\pi }{2}-\gamma ,\;\frac{3\pi }{2}+\gamma \right]\\ -\frac{\pi }{4\gamma } & \mathrm{on}\;\mathrm{the}\;\mathrm{electrode}\;B, & \theta \in \left[\frac{\pi }{2}-\gamma ,\;\frac{\pi }{2}+\gamma \right]\\ 0 & \mathrm{otherwise} & \end{array}\right.$$

Multiplying Equation (14) by cos(mθ) and solving the integral in the interval of [0, 2π]:

$$\pi ma^{m-1}C_{m}^{\prime }=0$$

hence,

(15) $$C_{m}^{\prime }=0$$

Similarly, multiplying Equation (14) by sin(mθ) and solving the integral in the interval of [0, 2π]:

(16) $$\pi ma^{m-1}C_{m}=\frac{\pi }{m\gamma }\sin(m\gamma )\sin(\frac{m\pi }{2})\cos(m\pi )$$

For $\underset{\gamma \to 0}{\lim}\frac{\sin\left(m\gamma \right)}{m\gamma }=1$, Equation (16) can be simplified as

(17) $$C_{m}=\frac{{\left(-1\right)}^{k}}{a^{m-1}m}\left(m=2k-1\right)$$

Substituting Equations (16) and (17) into Equation (13), *G* can be written as:

(18) $$\begin{array}{ll} G & =\sum_{k=1}^{\infty }r^{m}C_{m}\sin(m\theta )\\ & =\sum_{k=1}^{\infty }r^{m}\frac{{\left(-1\right)}^{k}\sin(m\gamma )}{a^{m-1}m^{2}\gamma }\sin(m\theta )\\ & =\;\sum_{k=1}^{\infty }\frac{{\left(-1\right)}^{k}a}{m}{\left(\frac{r}{a}\right)}^{m}\sin(m\theta )\left(m=2k-1\right) \end{array}$$

The weight function of the downhole inserted two-electrode EMF in the cylindrical coordinate system can be expressed as follows:

(19) $$W=\sum_{k=1}^{\infty }{\left(-1\right)}^{k}{\left(\frac{r}{a}\right)}^{m-1}\left[\sin\left(m\theta \right)e_{r}+\cos(m\theta )e_{\theta }\right]\;\left(m=2k\;-1\right)$$

To study the weight function distribution inside the flowtube, this work conducted a numerical calculation on Equation (19), and the results of the numerical calculation were visualized graphically.

Suppose r∈[0, a], *a* = 5.5 mm (*a* represents the inner radius of the EMF); the calculating step is Δr=0.1 mm. Suppose θ∈[0, 2π], the calculating step is Δθ=π360. 

**Figure 3:**
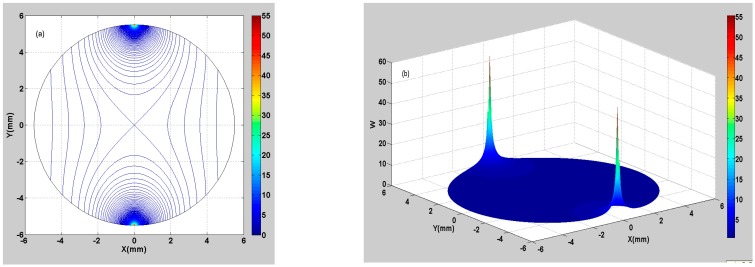
Weight function distribution of the downhole inserted EMF. (**a**) 2D contour plot of the weight function; (**b**) 3D surface plot of the weight function.

Figure 3a shows the 2D contour plot of the numerical calculation results of the weight function. Figure 3b shows the 3D surface plot of the numerical calculation results of the weight function. It is found that the maximum value of the weight function is 55.68 from the electrode positions, whereas the minimum value is 0.25 from the magnetic pole positions (the weight function is dimensionless). The color map changes from red to blue, representing a decrease in the weight function. On the whole, the weight function is relatively larger near the electrodes, indicating that the fluid elements in these regions have a great contribution to the potential difference. The weight function is relatively smaller in other regions, indicating that the fluid elements in these regions have a lower contribution to the potential difference.

### 2.3. Magnetic Field of the Two-Electrode EMF 

The magnetic field of the downhole inserted two-electrode EMF is formed by two current-carrying coils. Giving a direct and strict analytical formula of the magnetic induction intensity for the actual coil structure is difficult. Alternatively, the two current-carrying coils are equivalent to two magnetic dipoles, and according to the assumption of column symmetry, the magnetic induction intensity of the magnetic dipoles that is perpendicular to the axial direction can be simplified as a plane distribution.

The magnetic vector potential ***A*** is the superposition generated by the two magnetic dipoles—i.e.,

(20) $$A=\;A_{1}\;+\;A_{2}$$

where ***A***_1_ and ***A***_2_ are the magnetic vector potentials of the two magnetic dipoles. The magnetic induction of the magnetic dipoles can be expressed as:

(21) B=∇×A

**Figure 4:**
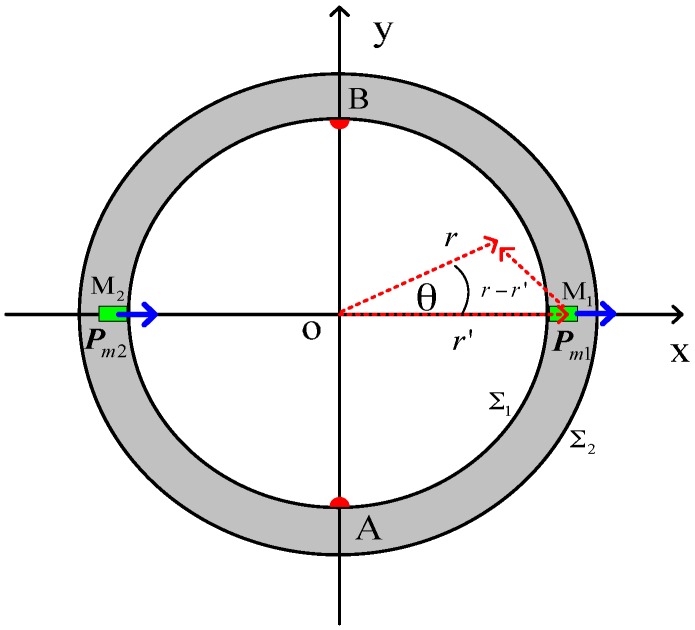
Schematic diagram of the magnetic dipoles of the downhole inserted EMF.

To solve the magnetic vector potential distribution of magnetic dipoles in the cylindrical coordinate systems, as shown in Figure 4, we assume that the two magnetic dipoles are marked as ***P***_m1_ and ***P***_m2_, whereas their coordinates are (r′, 0) and (r′, π) in the polar coordinate systems, respectively. ***P***_m1_ point in the *r* direction, whereas ***P***_m2_ points opposite to the *r* direction.

The magnetic vector potential distribution of ***P***_m1_ in space can be written as follows:

(22) $$A_{1}=\frac{1}{4\pi {\left|r-r\prime \right|}^{3}}P_{m1}\times (r-r\prime )$$

where $P_{m1}=P_{m}=\mu Is$, and μ, *I*, and *s* represent the coil magnetic permeability, the current intensity, and the coil area, respectively. ${\left|r-r\prime \right|}^{3}$can be expressed as:

(23) $${\left|r-r\prime \right|}^{3}={\left[r^{2}+d^{2}-2rd\cos(\theta )\right]}^{\frac{3}{2}}$$

where |r′|=d, and *d* is the radial position of the magnetic dipole. ***P***_m1_ is in the same direction as r′, such that

(24) $$P_{m1}\times r^{\prime }=0$$

(25) $$P_{m1}\times r=p_{m}r\sin(\theta )e_{z}$$

By substituting Equations (23)–(25) into Equation (22), the magnetic vector potential ***A***_1_ can be simplified as:

(26) $$A_{1}=\frac{p_{m}r\sin(\theta )}{4\pi {\left[r^{2}+d^{2}-2rd\cos(\theta )\right]}^{\frac{3}{2}}}e_{z}$$

Similarly, ***A***_2_ can be expressed as:

(27) $$A_{2}=\frac{-p_{m}r\sin(\theta )}{4\pi [{\left(r^{2}+d^{2}+2rd\cos(\theta )\right]}^{\frac{3}{2}}}e_{z}$$

By substituting Equations (26) and (27) into Equation (20) and, finally, into Equation (21), we derive

(28) $$\begin{array}{l} B=\frac{p_{m}}{4\pi }\left\{\begin{array}{l} -3dr^{2}{\left[r^{2}+d^{2}-2rd\cos(\theta )\right]}^{-\frac{5}{2}}\sin^{2}(\theta )+r{\left[r^{2}+d^{2}-2rd\cos(\theta )\right]}^{-\frac{3}{2}}\cos(\theta )\\ -3dr^{2}{\left[r^{2}+d^{2}+2rd\cos(\theta )\right]}^{-\frac{5}{2}}\sin^{2}(\theta )-r{\left[r^{2}+d^{2}+2rd\cos(\theta )\right]}^{-\frac{3}{2}}\cos(\theta ) \end{array}\right\}e_{r}\\ -\frac{rp_{m}}{4\pi }\sin(\theta )\left\{\begin{array}{l} {\left[r^{2}+d^{2}-2rd\cos(\theta )\right]}^{-\frac{3}{2}}-3r[r-d\cos(\theta )]{\left[r^{2}+d^{2}-2rd\cos(\theta )\right]}^{-\frac{5}{2}}+\\ -{\left[r^{2}+d^{2}+2rd\cos(\theta )\right]}^{-\frac{3}{2}}+3r[r+d\cos(\theta )]{\left[r^{2}+d^{2}+2rd\cos(\theta )\right]}^{-\frac{5}{2}} \end{array}\right\}e_{\theta } \end{array}$$

In addition, to study the magnetic field distribution inside the flowtube, this work conducted a numerical calculation on Equation (28), and the results of the numerical calculation were visualized graphically.

The ranges of variables *r*, *θ*, and the calculating step are the same as in Equation (19). Suppose *d* = 9.5 mm (*d* represents the distance between the center of the flowmeter and the magnetic dipole), and *p_m_* = 0.01 H·A·m (*p_m_* is an intermediate variable). 

**Figure 5:**
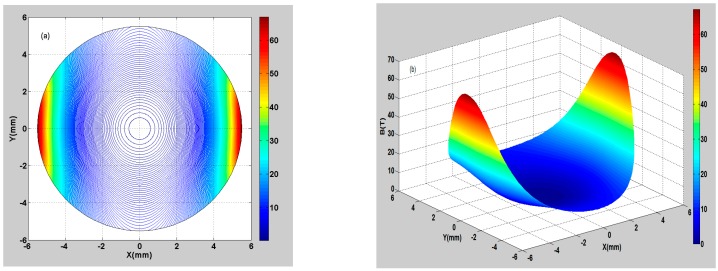
Magnetic field distribution of the downhole inserted EMF. (**a**) 2D contour plot of the magnetic field; (**b**) 3D surface plot of the magnetic field.

Figure 5a displays the 2D contour plot of the numerical calculation results of the magnetic field. Figure 5b is the 3D surface plot of the numerical calculation results of the magnetic field. It is seen that the maximum value of the magnetic field is 65.36 from the magnetic pole positions, whereas the minimum value is 0.06 from the electrode positions. The color map changes from red to blue, representing the decrease in the magnetic field. In general, the magnetic field inside the flowtube is relatively larger close to the magnetic poles, indicating that the magnetic field in these regions makes a larger contribution to the potential difference. The values of the magnetic field are relatively smaller in the other positions, indicating that the magnetic field in these regions has a smaller contribution to the potential difference. It can be concluded that the feature of the magnetic field distribution can improve the measurement sensitivity of the sensor and is beneficial for the two-electrode EMF to measure the flowrate. 

## 3. Dynamic Experiments on Multiphase Flow Loop Facility

### 3.1. The Downhole Inserted Two-Electrode EMF

**Figure 6:**
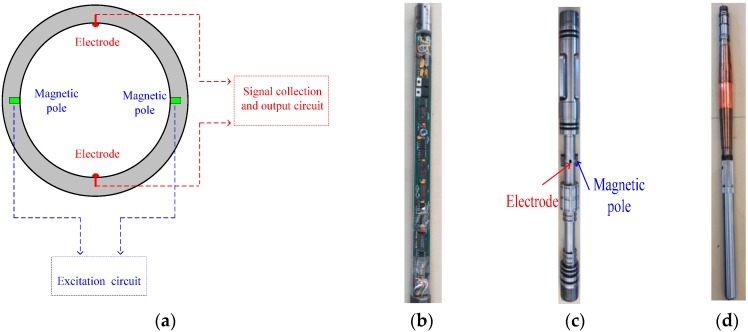

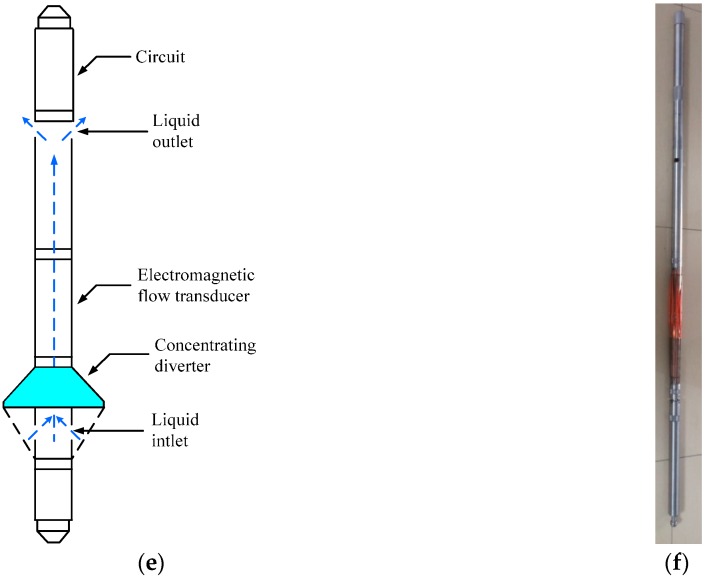
Diagrams of the downhole inserted EMF. (**a**) The diagram of the measuring principle of the EMF; (**b**) Photo of the measuring circuit; (**c**) Photo of the transducer; (**d**) Photo of the concentrating diverter; (**e**) Diagram of the downhole EMF; (**f**) Photo of the downhole EMF.

Figure 6a shows a diagram of the measuring principle of the EMF, the excitation circuit producing the magnetic field, and the signal collection circuit measuring the induction electromotive force, which is converted to a frequency signal through the signal output circuit; hence, the response frequency of the transducer is proportional to the flowrate. Figure 6b shows a photo of the EMF measuring circuit, Figure 6c shows a photo of the EMF transducer, Figure 6d shows a photo of the concentrating diverter, Figure 6e shows a diagram of the downhole EMF, and Figure 6f shows a photo of the downhole EMF.

The measurement method of the downhole inserted EMF can be described as follows: The flowmeter contains a petal type concentrating diverter (PTCD) and electromagnetic flow transducer circuit. When the flowmeter starts operation, it first opens the PTCD to seal the annular space between the flowmeter and the boring casing, which forces oil-water mixture fluid to flow into the measurement channel through the liquid inlet. The fluid then flows vertically upward through the electromagnetic flow transducer and the liquid outlet in series. The PTCD can enhance the homogeneous degree of the oil-water mixture by accelerating the fluid velocity and reduce the influence of oil-water slippage on the transducer output. The PTCD also functions as a centralizer, which ensures that the flowmeter is located at the central position in the boring casing, thus effectively reducing measurement errors.

### 3.2. Experimental Design

The experimental conditions comprised normal pressure and temperature, and the experimental media were diesel oil and tap water. The total flowrates of oil-water two-phase flow ranged from 2 to 60 m^3^/d. The water-cut was from 50% to 100%. For a specific experimental process, the oil-water two-phase total flowrate was first set, and then the water-cut was adjusted by a step of 10%. Finally, when the flow stabilized, the response values of the electromagnetic flow transducer were recorded.

A total sampling time of 180 s was applied at intervals of 0.5 s under each flow condition. Thus, 360 response values were recorded for each flow condition, and the average value was used as the corresponding measurement value. 

### 3.3. Experimental Setup

The large experimental vertical simulation well facility for oil-water two-phase flow is located at the Daqing Production Well Logging Institute (DPWL) in Heilongjiang Province, China. The facility consists of a transparent Plexiglas wellbore, two overhead flow-stabilizing towers, an oil-water separation tank, and flowrate adjustment devices.

**Figure 7:**
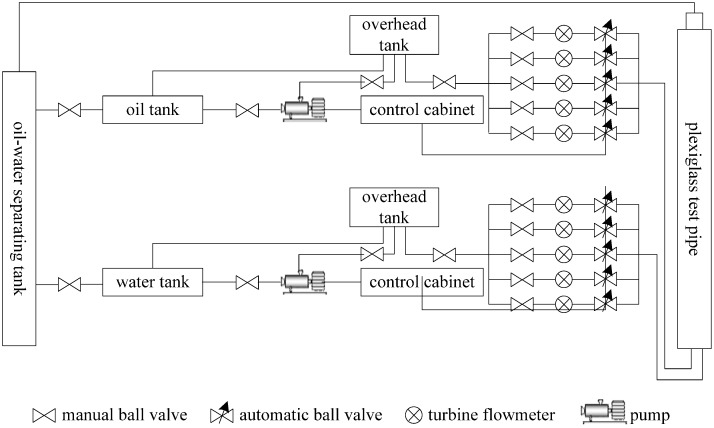
The diagram of the vertical simulation well facility.

Figure 7 shows a schematic of the operating process for the facility. Tap water and diesel oil are separated and purified before being pumped into tanks on the overhead flow-stabilizing towers, which are 45 m in height. Steady flowrates are maintained by a constant liquid level in the tanks. Overflow valves installed in the tanks ensure constant diesel oil and tap water levels. Diesel oil and tap water overflows are directed through these valves to storage tanks on the ground. 

Diesel oil or tap water from the tanks on the stabilizing towers flows into the wellbore at the bottom of a transparent Plexiglas pipe (125 mm in inner diameter, 26 m in length). Intensive mixing of diesel oil and tap water is ensured by the length of the simulation wellbore.

The flowrate of diesel oil and tap water can be accurately controlled and metered using adjustment devices and measuring instruments. The EMF was located at the upper portion of the simulation wellbore. Oil-water two-phase fluid flowed into the two-phase separation tank, and diesel oil and tap water were separated and recycled. Experimental work on single-phase flow and two-phase flow can be carried out in this experimental facility.

### 3.4. Response Characteristics of the Two-Electrode EMF in Oil-Water Two-Phase Flow

**Figure 8:**
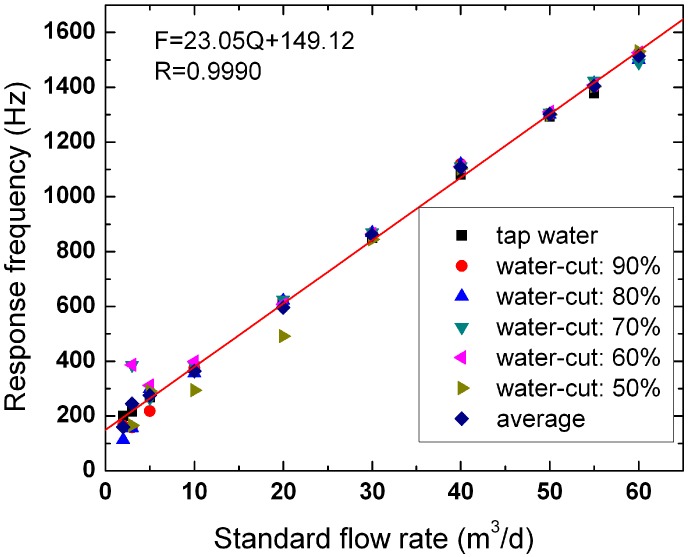
The linear fitting chart of EMF No. 1.

**Figure 9:**
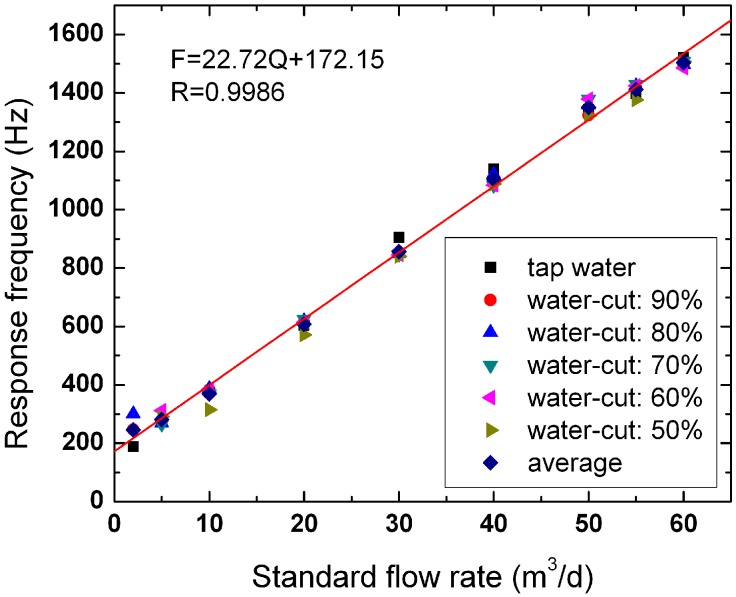
The linear fitting chart of EMF No. 2.

We conduct experiments on oil-water two-phase flow by EMFs No. 1 and No. 2 in the multiphase flow loop facility. We also study the response characteristics of the EMFs in oil-water two-phase flow. Figure 8 and Figure 9 show the linear fitting chart of EMFs No. 1 and No. 2, respectively. The abscissa and ordinate represent the standard flowrate and instrument response frequency, respectively. The standard flowrate represents the flowrate controlled and metered by the adjustment devices and measuring instruments under each flow condition. For each standard flowrate, we calculate the average response frequency of six flow conditions and then conduct linear fitting on the average response frequency (as shown in Figure 8 and Figure 9 by the red line). The correlation coefficients of the two EMFs are 0.9990 and 0.9986, respectively.

**Figure 10:**
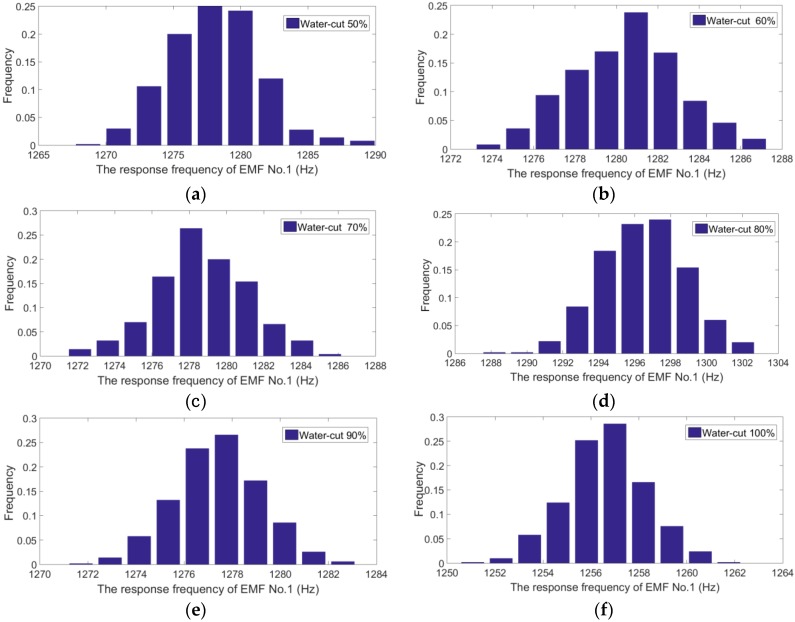
The measurement data *x_i_* histogram of EMF No. 1 (flowrate: 50 m^3^/d). (**a**) The histogram for water-cut 50%; (**b**) The histogram for water-cut 60%; (**c**) The histogram for water-cut 70%; (**d**) The histogram for water-cut 80%; (**e**) The histogram for water-cut 90%; (**f**) The histogram for water-cut 100%.

According to literatures [19,20], we analyze the measurement data. We find that the measurement data obey Gaussian behavior. An example has been added into the manuscript, as shown in Figure 10. The abscissa and ordinate represent the response frequency of EMF No. 1 and frequency, respectively. Figure 10a–f shows the distribution of the EMF No. 1 measurement data for different water-cuts at a constant flowrate of 50 m^3^/d. The mathematical expectations are 1278 Hz, 1280 Hz, 1279 Hz, 1296 Hz, 1278 Hz, and 1257 Hz, respectively. The variances are 11.6 Hz, 6.7 Hz, 6.2 Hz, 5.7 Hz, 3.2 Hz, and 2.8 Hz, respectively.

For each flow condition, outliers are treated and excluded by using Laiyite criterion (i.e., 3δ criterion) as follows:

(1) Calculating the average value of the measurement data by Equation (29),
  (29) $$\bar{x}=\frac{1}{360}\sum_{i=1}^{360}x_{i}$$
  where $x_{i}$ and $\bar{x}$ represent the response frequency and the average response frequency of the electromagnetic flow transducer, respectively.
(2) Calculating the standard deviation of the measurement data by Equation (30),
  (30) $$\delta =\sqrt{\frac{\sum_{i=1}^{n=360}{\left(x_{i}-\bar{x}\right)}^{2}}{n-1}}$$
  where δ represents the standard deviation.
(3) If $\left|x_{i}-\bar{x}\right|\rangle 3\delta$, $x_{i}$ are treated outlier and excluded. Then, the average value and the standard deviation are re-calculated, respectively.

According to literatures [19,20], we evaluated the linear correlation of the measurement data by using ANOVA (Analysis of variance) method. 

Table 1 and Table 2 list the ANOVA (Analysis of variance) of EMFs No. 1 and No. 2, respectively. 

As listed in Table 1, the value of F-test of SS_LOF_ (1.02) is less than F_0.1_ (1.83), indicating that the F-test of SS_LOF_ is not significant. Furthermore, the LOF can be neglected relative to the experimental error. Based on ANOVA method, the measurement data of EMF No. 1 are highly linearly correlated, while the measurement data of EMF No. 1 are not affected by the water-cuts. 

Similarly, the ANOVA results listed in Table 2 indicate the measurement data of EMF No. 2 are also highly linearly correlated, and the measurement data of EMF No. 2 are also not affected by the water-cuts.

Table 3 and Table 4 show the full-scale errors of EMFs No. 1 and No. 2 for each flow condition, respectively. The full-scale error can be calculated by Equations (31) and (32).

(31) $$\bar{X}=\sum_{m=1}^{6}{\bar{x}}_{m}$$

where ${\hat{x}}_{m}$ and $\bar{X}$ represent the response frequency for each water-cut and the average response frequency, respectively.

(32) $$r_{F}=\frac{{\bar{x}}_{m}-\bar{X}}{60}\times 100\%$$

where $r_{F}$ represents the full-scale error corresponding to each flow condition.

Table 3 lists the full-scale errors of EMF No. 1 corresponding to each flow condition. 

Table 4 lists the full-scale errors of EMF No. 2 corresponding to each flow condition. 

Table 3 and Table 4 show that full-scale errors of the two EMFs are very similar for each flow condition. The errors are smaller when the flowrate is relatively high; the errors are larger when the flowrate is relatively low, which is attributed to the homogenization degree of the oil-water two-phase flow being high at the high flowrate, leading to smaller errors. Meanwhile, the homogenization degree of the oil-water two-phase flow deteriorates seriously, and the oil-water slippage phenomenon appears at the low flowrate; thus, the response signal fluctuates greatly because of the adhesive retention of the oil bubbles on the measuring electrodes, leading to larger errors. Therefore, the error analysis results of the dynamic experiments indicate that the EMF can be used for flowrate measurement of oil-water two-phase flow in the high-water-cut condition. The maximum absolute value of the full-scale errors is better than 5%, with a total flowrate of 5–60 m^3^/d and water-cut higher than 60%. The maximum absolute value of the full-scale errors is better than 7%, with a total flowrate of 2–60 m^3^/d and water-cut higher than 70%.

## 4. Onsite Experiments

To evaluate the application performance of the downhole inserted two-electrode EMF in oil-producing wells in Daqing Oilfield, the experimental results of two typical wells are introduced. 

X-** is a water flooding development well that has four perforated zones, whose wellhead production rate and water-cut are 51.2 m^3^/d and 86%, respectively. We tested the production rate at five depths by using the turbine flowmeter and EMF No. 1, respectively. The tested results are listed in Table 5.

In onsite experiments, many factors can result in measurement errors due to the complex downhole environments, including damage to the PTCFD; casing deformation and dislocation; solid floc winding of the turbine bearing, leading to variation of the instrument constant; solid particle blockage of the moving parts of the turbine flowmeter; etc. The flowrate measurements of the wellhead production rate of the turbine flowmeter at the depth of 1083.4 m, repeated three times, are significantly lower than 51.2 m^3^/d, with a minimum absolute error of 16.3 m^3^/d and a minimum absolute relative error of 31.84%. It is believed that the possible reason for the larger errors of the turbine flowmeter in the X-** well is solid floc winding of the turbine bearing, leading to variation of the instrument constant. The thrice-repeated flowrate measurements of EMF No. 1 are close to the wellhead production rate, the maximum absolute error is 1.1 m^3^/d, and the maximum absolute relative error is 2.15%. The thrice-repeated flowrate measurements of EMF No. 1 at depths of 1095.9 m, 1100.7 m, and 1106.6 m are very similar, indicating that the EMF can provide accurate measurements compared with the turbine flowmeter.

Y-** is a polymer flooding development well that has four perforated zones, and the wellhead production rate, water-cut, and viscosity are 38.5 m^3^/d, 77%, and 61.0 mPa·s, respectively. The tested results are listed in Table 6.

When we first tested the production rate by using the turbine flowmeter, a zero flowrate could be obtained at depths of 1030.3 m, 1036.7 m, 1045.2 m, and 1055.0 m, indicating failure of the testing. The viscosity of the output fluid was high, leading to the sum of the viscous friction resistance moment and mechanical friction resistance moment of the turbine flowmeter having overwhelming superiority relative to the driving moment. Hence, the turbine flowmeter did not rotate, and the readings displayed a zero flowrate. Furthermore, we tested the production rate by using EMF No. 2. The thrice-repeated flowrate measurements of the wellhead production rate of EMF No. 2 at a depth of 1030.3 m are significantly close to 38.5 m^3^/d. The minimum absolute error is 0.7 m^3^/d, and the minimum absolute relative error is 1.82%. 

Therefore, it is indicated that the EMF can accurately measure the actual production rate of oil wells. Particularly, compared with the conventional turbine flowmeter, the EMF has neither moving parts nor choke components. Hence, the EMF can greatly improve the success rate of well logging for these high-viscosity polymer flooding oil-producing wells.

## 5. Conclusions

The following conclusions can be drawn according to the above-mentioned analysis:

(1) The measurement principle, the weight function, and the magnetic field of the novel downhole inserted EMF are described.
(2) Dynamic experiments on two EMFs in oil-water two-phase flow are carried out, and the experimental errors are analyzed in detail. The data analysis results of the dynamic experiments show that the EMF can be used for flowrate measurement of oil-water two-phase flow for the high-water-cut condition.
(3) Furthermore, onsite experiments in high-water-cut oil-producing wells are conducted. The possible reasons for the errors in onsite experiments are analyzed. The results indicate that the EMF can provide an effective technology for measuring downhole oil-water two-phase flow.

## Figures and Tables

**Table 1:** The ANOVA table for EMF No. 1.

Source of Variation	Sum of Squares (Hz^2^)	Degrees of Freedom	Variance (Hz^2^)	F	Significance
Regression, REG	SS_REG_: 14,670,981.14	1	14,670,981.14	9138.56	F_0.01_: 7.31
Lack of fit, LOF	SS_LOF_: 28,333.51	8	3541.69	1.02	F_0.01_: 2.99
					F_0.1_: 1.83
Pure error, PE	SS_PE_: 173,091.59	50	3461.83	-	-
Total	SS_T_: 14,779,584.29	59	-	-	-

**Table 2:** The ANOVA table for EMF No.2.

Source of Variation	Sum of Squares (Hz^2^)	Degrees of Freedom	Variance (Hz^2^)	F	Significance
Regression, REG	SS_REG_: 12,182,370.25	1	12,182,370.25	2934.80	F_0.01_: 7.31
Lack of fit, LOF	SS_LOF_: 33,009.10	7	4715.59	1.14	F_0.01_: 3.29
					F_0.1_: 1.83
Pure error, PE	SS_PE_: 186,795.50	45	4151.01	-	-
Total	SS_T_: 12,402,174.85	53	-	-	-

**Table 3:** The full-scale errors of EMF No. 1.

	Water-Cut	100%	90%	80%	70%	60%	50%
Flowrate							
60 m^3^/d		0.01%	0.70%	−1.06%	−1.90%	0.84%	1.17%
55 m^3^/d		−1.72%	0.03%	0.93%	1.62%	0.47%	0.08%
50 m^3^/d		0.27%	0.94%	0.93%	1.33%	1.57%	0.79%
40 m^3^/d		1.40%	4.21%	4.20%	3.60%	3.56%	3.00%
30 m^3^/d		1.09%	2.36%	2.48%	2.49%	2.08%	0.61%
20 m^3^/d		−0.03%	0.79%	0.92%	1.13%	−0.14%	−8.72%
10 m^3^/d		−0.48%	−0.53%	−1.99%	0.14%	1.13%	−6.57%
5 m^3^/d		−0.13%	−3.78%	2.18%	−0.33%	−4.50%	5.93%
3 m^3^/d		−0.62%	−4.91%	−5.03%	11.84%	11.98%	−4.35%
2 m^3^/d		−0.11%	−2.73%	−6.48%	18.37%	19.26%	−40.78%

**Table 4:** The full-scale errors of EMF No. 2.

	Water-Cut	100%	90%	80%	70%	60%	50%
Flowrate							
60 m^3^/d		−2.87%	−2.56%	−2.44%	−3.08%	−2.27%	−3.86%
55 m^3^/d		−3.36%	−2.05%	−1.42%	−1.09%	−1.43%	−4.90%
50 m^3^/d		1.06%	1.86%	2.17%	3.57%	3.65%	−0.52%
40 m^3^/d		3.24%	4.29%	4.10%	−0.73%	−0.66%	−0.44%
30 m^3^/d		3.19%	−0.70%	−0.74%	−0.89%	−1.01%	−1.38%
20 m^3^/d		−1.61%	−0.84%	−0.46%	−0.09%	−0.78%	−3.86%
10 m^3^/d		−1.12%	−0.67%	−0.28%	−1.55%	−0.50%	−5.48%
5 m^3^/d		−0.19%	0.05%	−0.36%	−0.65%	2.64%	1.17%
2 m^3^/d		−1.17%	3.02%	6.86%	25.54%	30.39%	47.88%

**Table 5:** The contrast logging results of EMF No. 1 and the turbine flowmeter in the X-** well.

Test Depth (m)	Perforated Zone	Turbine Flowmeter			No. 1 EMF		
		First (m^3^/d)	Second (m^3^/d)	Third (m^3^/d)	First (m^3^/d)	Second (m^3^/d)	Third (m^3^/d)
1083.4	X1	34.6	34.9	34.9	52.2	52.2	52.3
1095.9	X2	13.9	14.1	14.0	22.5	22.5	22.2
1100.7	X3	5.4	5.6	5.8	10.8	10.4	10.6
1106.6	X4	2.1	2.4	2.2	5.2	5.5	5.8
1180.0	bore-hole bottom	0	0	0	0	0	0

**Table 6:** The contrast logging results of EMF No. 2 in the Y-** well.

Test Depth (m)	Perforated Zone	Turbine Flowmeter			No. 2 EMF		
		First (m^3^/d)	Second (m^3^/d)	Third (m^3^/d)	First (m^3^/d)	Second (m^3^/d)	Third (m^3^/d)
1030.3	Y1	0	0	0	37.0	37.3	37.8
1036.7	Y2	0	0	0	15.7	15.5	15.1
1045.2	Y3	0	0	0	10.8	10.4	10.6
1055.0	Y4	0	0	0	7.4	7.1	7.8
1139.9	bore-hole bottom	0	0	0	0	0	0
